# Augmented reality in clinical skills simulation: usability and feasibility of smart glasses in a simulated environment

**DOI:** 10.1590/acb414626

**Published:** 2026-08-03

**Authors:** Carlos Edmundo Rodrigues Fontes, Rafael Fernandez Castilio, Félix Plaza Moreno, Mateus de Amorim Aboboreira, Maria Luíza Bernardo de Lima, Yves Henrique Ramos Mansano, Claudio Bodgan, Willian Cesar Cavazana, Miyoko Massago, Sanderland José Tavares Gurgel, Luciano de Andrade

**Affiliations:** 1 Universidade Estadual de Maringá – Department of Medicine – Maringa (PR), Brazil.; 2 Universidad de Granada – Faculty of Health Sciences – Granada, Spain.; 3 Hospital Universitário Regional de Maringá – Maringa (PR), Brazil.; 4 Universidade Estadual de Maringá – Department of Health Sciences – Maringa (PR), Brazil.

**Keywords:** Patient Simulation, Smart Glasses, Simulation Training, Augmented Reality, Telemedicine

## Abstract

**Purpose:**

To assess the usability and feasibility of augmented-reality smart glasses during simulated pediatric bladder catheterization with real-time remote specialist guidance.

**Methods:**

Thirty-five critical-care graduate healthcare professionals simulated pediatric bladder catheterization on a manikin using Vuzix M400 glasses via telemedicine. After brief familiarization, participants completed a seven-item Likert (0–7) questionnaire covering ease of use, procedural understanding, confidence/efficiency, comfort, and global acceptance. We analyzed descriptive statistics, internal consistency (Cronbach’s α), and item-domain coherence. A conceptual diagram and synthesis table complemented quantitative data.

**Results:**

Augmented-reality-guided training showed generally favorable usability perceptions. Median scores ranged 3.5–5, with strongest ratings for observation support, image quality, procedural understanding, and intention to recommend. Comfort and efficiency scored lower (median = 3.5), indicating more neutral perceptions. The instrument showed high internal consistency (α = 0.97), although this may reflect item redundancy, and item-domain alignment provided a descriptive representation of the conceptual model.

**Conclusion:**

These findings suggest that augmented-reality smart glasses are feasible and generally well accepted for simulated remotely guided training. Further controlled studies with objective performance measures are needed.

## Introduction

Augmented reality (AR) has emerged as a promising technology in healthcare education, enabling the integration of digital information into real-world clinical environments^1^. Internationally, AR has been applied in a variety of educational contexts, including surgical training, anatomical visualization, procedural guidance, and remote telementoring^2-4^. These applications have been associated with potential benefits such as improved visualization, enhanced learner engagement, and the possibility of real-time expert support^5,6^.

Despite these advances, the current literature remains heterogeneous and is still largely characterized by exploratory studies with insufficiently detailed implementation frameworks^7^. Several studies are limited to controlled environments, small-scale implementations, or non-interactive systems, with relatively few investigations exploring real-time, remote-guided applications using wearable devices such as smart glasses^5-7^. Furthermore, despite increasing international adoption of AR in healthcare education, evidence regarding usability and feasibility in practical training scenarios remains limited, particularly in settings involving distributed teams and telemedicine integration.

Most existing AR research focuses on complex surgical procedures or advanced simulations, leaving limited evidence on how immersive technologies influence practical learning in routine clinical tasks or in basic procedural training, areas where learners frequently struggle and iatrogenic errors are common^8^. This gap is particularly relevant in the context of scalable and accessible training strategies across different healthcare systems.

Bladder catheterization is a common yet technically demanding clinical procedure that requires adequate training to ensure patient safety^9^. The procedure involves strict adherence to aseptic technique, precise anatomical knowledge, and careful handling to avoid complications such as infection, urethral trauma, or incorrect placement^10^. Traditional teaching methods often rely on direct supervision and repetition in clinical or simulated settings, but providing consistent, real-time expert guidance can be challenging, especially in resource-limited or remote environments. These challenges make it a relevant model for evaluating technologies aimed at supporting procedural learning and supervision.

Wearable devices such as smart glasses offer hands-free operation, real-time projection of procedural steps, and improved situational awareness, making them promising tools to support guided practice in both simulation and clinical environments^11,12^. However, studies also report challenges related to ergonomics, device comfort, and integration into clinical workflows, underscoring the need for usability evaluations across different contexts and levels of procedural complexity^13,14^.

From this perspective, AR-enabled smart glasses offer a potential solution by allowing healthcare professionals to receive visual guidance and expert support without interrupting procedural workflow^15^. Their integration with telemedicine may enable remote-assisted training and supervision across geographically distributed settings, which remains underexplored in the literature. This hands-free technology may be particularly valuable in simulation-based education, in which controlled environments can be used to evaluate usability and user’s experience prior to broader implementation.

Within this framework, investigating AR-supported guidance in common procedures, such as pediatric bladder catheterization, may contribute valuable evidence to inform medical education practices and simulation training in Brazil and other healthcare settings^16^. Therefore, the present study aimed to evaluate the usability and feasibility of AR smart glasses during a simulated bladder catheterization procedure supported by real-time remote guidance. This work focused on user’s perception and feasibility within a simulated, tele-assisted context, providing initial insights to support future research on the clinical and training-related applications of AR technologies.

## Methods

### Study design

This study was designed as a single-group, post-intervention usability, and feasibility study conducted in a simulated clinical setting. A simulation-based procedure training scenario was used to assess the usability of AR smart glasses (Vuzix M400, Vuzix Corporation, Rochester, NY, United States of America) by healthcare professionals during a simulated pediatric bladder catheterization. To ensure participant safety, all activities were performed in a controlled hospital simulation environment.

The study protocol received ethical approval from the Research Ethics Committee of the Universidade Estadual de Maringá (CAAE 82464724300000104), Brazil, and formally included collaboration with the Universidad de Granada, Spain. In alignment with this approved international partnership, the simulations were conducted at the Universidad de Granada during the first author’s postdoctoral training.

### Participants

Thirty-five healthcare professionals enrolled in a Master’s program in Critical Care at the Universidad de Granada were included. Eligibility criteria included: at least one year of clinical experience, absence of severe visual impairment, and no prior experience with smart glasses. Participants with varying levels of prior experience in bladder catheterization were recruited.

### Simulation scenario

The simulation was based on a clinical case of an 8-month-old male infant presenting with fever, irritability, and decreased urine output, with a history of urinary tract infection. Bladder catheterization was indicated to obtain a sterile urine sample and relieve urinary retention. The procedure was performed on a Laerdal pediatric manikin representing the patient. The simulation environment included a stable internet connection, and a 360° camera was used exclusively to document the simulation environment and provide contextual visualization, without interfering with the remote guidance process.

### Intervention and procedures

Before the simulation, all participants underwent a 10-minute familiarization session, which included instructions on device operation, interface navigation, and activation of AR functionalities. No formal competency assessment was performed prior to the procedure.

**Figure 1:**
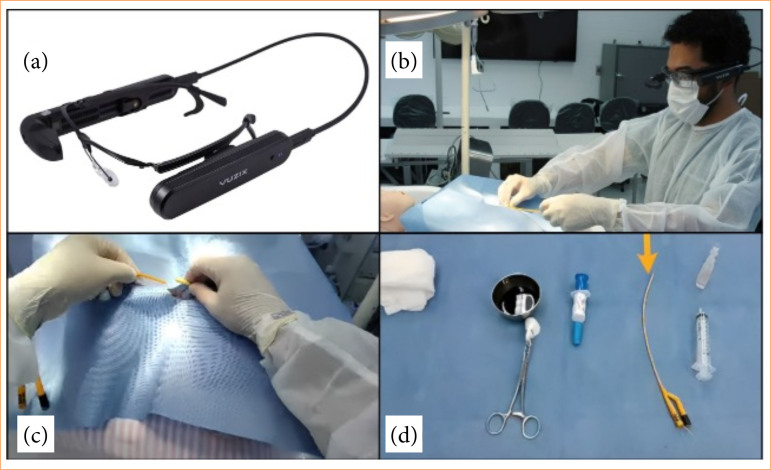
Vuzix M400 augmented-reality smart glasses and their use during the simulated pediatric bladder catheterization with real-time telementoring. (a) Smart glasses with external battery module and main device components, including a 12.8-MP camera capable of transmitting high-resolution video for remote supervision. (b) Participant wearing the smart glasses during the procedure while receiving step-by-step remote guidance. (c) First-person view captured by the smart glasses, illustrating the hands-free visualization transmitted to the remote specialist. (d) Instrument table prepared for the simulation, with a yellow arrow highlighting the pediatric Foley catheter, an example of visual emphasis used to simulate augmented-reality guidance during telementoring.

Source: Elaborated by the authors.

During the simulation, participants performed a catheterization procedure on a pediatric manikin while wearing AR smart glasses. A single remote specialist provided real-time guidance through a telemedicine platform, using the point-of-view video transmitted by the device. The specialist provided step-by-step visual and auditory instructions. These included patient positioning, aseptic preparation, catheter selection and lubrication, insertion depth, connection to the drainage system, and safe catheter removal. The specialist monitored the procedure in real time through the glasses’ camera, offering immediate feedback and corrections when necessary (Fig. 1).

### Data collection and analysis

Usability was assessed through a custom questionnaire inspired by conceptual elements of the system usability scale (SUS) and the technology acceptance model (TAM). Given the exploratory nature of this pilot study, the instrument was designed to capture context-specific usability perceptions relevant to the simulated AR setting. Responses were recorded using a 0–7 Likert scale without a neutral midpoint, to encourage directional responses and minimize central tendency bias. The instrument consisted of seven questionnaire items, as detailed in Table 1, each ranging from 0 (“strongly disagree”) to 7 (“strongly agree”).

**Table 1:** Items included in the usability questionnaire administered at the end of the simulation, identification codes, conceptual domains, and one global acceptance item.

Code	Item statement	Conceptual domain
p1	The use of smart glasses facilitated the observation of the procedure.	D1 – Ease of use
p2	The image quality provided by the smart glasses was adequate.	D1 – Ease of use
p3	The smart glasses improved my understanding of the bladder catheterization procedure.	D2 – Learning / procedural understanding
p4	The use of smart glasses increased my confidence in performing the procedure.	D3 – Confidence / Efficiency
p5	The bladder catheterization procedure was more efficient with the use of smart glasses.	D3 – Confidence / efficiency
p6	The smart glasses were comfortable to use throughout the entire observation.	D4 – comfort
p7	I would recommend the use of smart glasses for similar medical procedures.	Global acceptance (single item)

Source: Elaborated by the authors.

The questionnaire was designed by the research team to address the specific characteristics of the simulated environment using AR smart glasses. Its development followed a structured process that included expert review, a pilot clarity assessment, and theoretical alignment with established usability and educational technology frameworks. First, an initial pool of items was drafted by the investigators and reviewed by two specialists in medical education and clinical simulation to ensure content validity, clarity, and relevance. Next, the instrument underwent a brief pre-test for clarity with a small group of graduate students not included in the final sample; minor wording adjustments were made based on this feedback, and no pilot responses were included in the analysis. Although the questionnaire was not adapted from a previously validated scale, item formulation was guided by principles derived from the SUS^14^ and the TAM^17^, particularly regarding perceived ease of use, usefulness, efficiency, and user’s satisfaction.

The final version of the instrument was structured into four conceptual domains relevant to AR-supported clinical simulation. These domains were defined for descriptive and organizational purposes and should not be interpreted as a validated factorial structure. Domain 1 (ease of use) included items related to device clarity, intuitiveness, and visual support (p1 and p2). Domain 2 (learning / procedural understanding) comprised the item assessing the extent to which participants perceived improved procedural understanding of the catheterization steps (p3). Domain 3 (confidence / efficiency) included items capturing perceived improvements in confidence and procedural efficiency when using the device (p4 and p5). Domain 4 (comfort) reflected ergonomic aspects experienced during device use (p6). In addition, a global single-item measure (p7) assessed overall acceptance and intention to recommend the technology. This structure is reflected in Table 1 and corresponds to the conceptual organization presented in the results section.

Non-responses were excluded from the dataset. Descriptive statistics were calculated, including medians and interquartile ranges. Internal consistency was assessed using Cronbach’s alpha and item–total correlations were calculated to support the internal consistency analysis. All analyses were conducted using R software. No objective performance metrics were collected, as the study focused exclusively on perceived usability and user’s experience.

### Likert-scale responses and internal consistency

Likert-scale responses were examined descriptively, and bar charts were used to visualize response distributions. Internal consistency of the seven-item questionnaire was assessed using Cronbach’s alpha with 95% confidence intervals, and average inter-item correlations.

### Visual modeling of item-domain relationships

A visual model was developed to illustrate how the questionnaire items mapped onto their predefined conceptual domains. The diagram followed a hierarchical structure with a central node representing overall usability, intermediate nodes for the four domains, and terminal nodes for the individual items. The domains were defined as:

- D1: ease of use (p1, p2);
- D2: learning / procedural understanding (p3);
- D3: confidence / efficiency (p4, p5);
- D4: comfort (p6).

The global acceptance item (p7) was represented separately.

Given the sample size (n = 35), a descriptive approach based on classical test theory^18^ was used. Item–total correlations were computed to indicate the strength of association between each item and its corresponding domain. Domain and global scores were derived to support the construction of the diagram.

This visualization provides a concise overview of the internal structure of the instrument, offering a descriptive representation of how items relate to their conceptual domains and how these domains organize participants’ overall assessment of the smart glasses.

### Conceptual synthesis of findings

To complement the quantitative results, a structured conceptual synthesis was developed to explore the potential implications of smart glasses use during the simulated procedure. This synthesis organized the findings into predefined conceptual dimensions (procedural understanding, learner confidence, visual clarity, engagement and motivation, efficiency, and comfort). The goals were to integrate numerical results with their possible relevance for clinical skills simulation and to identify theoretical hypotheses and anticipated applications that may be explored in future research. This summary is presented in Chart 1 of the Results section.

## Results

### Descriptive analysis

**Figure 2:**
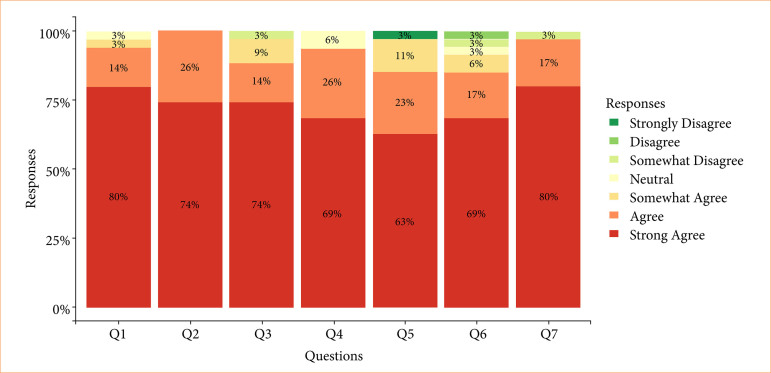
Distribution of responses across questionnaire items. Stacked bar chart illustrating the frequency of Likert-scale responses (0 = strongly disagree to 7 = strongly agree) for each of the seven usability questions. Most responses fell within the highest agreement categories (agree and strongly Agree, shown in orange and red), indicating overall positive acceptance of the smart glasses.

Source: Elaborated by the authors.

Most responses fell within the positive agreement categories (“agree” or “strongly agree”) across all items. The distribution of Likert-scale responses for each item is presented in Fig. 2. Median responses ranged from 3.5 to 5 on a 0–7 scale. Items related to procedural facilitation, image quality, and understanding showed higher median values (up to 5), while items related to comfort and efficiency presented median values of 3.5, corresponding to neutral perceptions. These findings indicate generally favorable but heterogeneous user’s perceptions, rather than uniformly high acceptance.

### Internal consistency

The questionnaire showed high internal consistency (Cronbach’s α = 0.97). However, the high mean inter-item correlation (r = 0.86) suggests potential redundancy among items.

### Item-domain relationships

**Figure 3:**
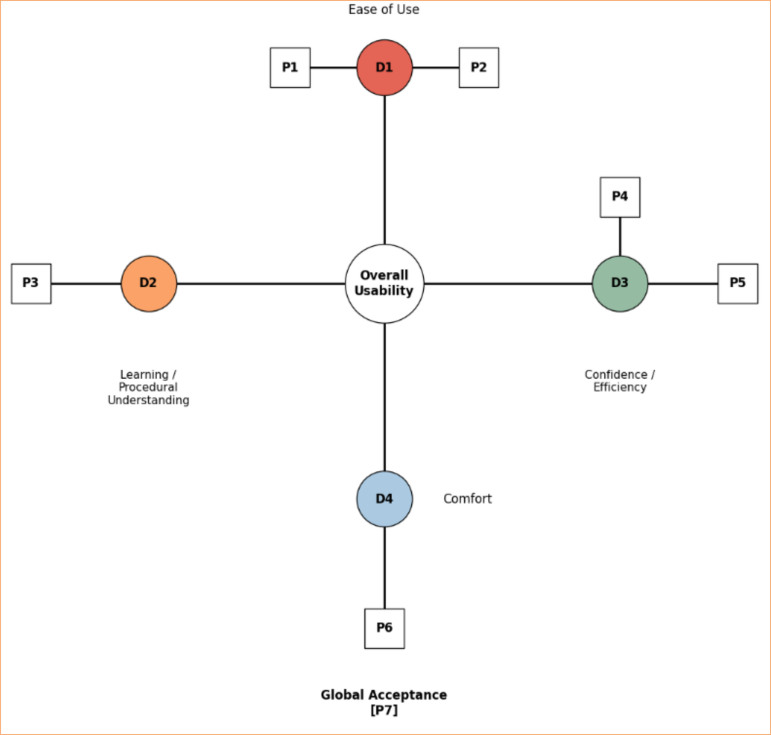
Conceptual diagram illustrating item–domain relationships.

Source: Elaborated by the authors.

A descriptive examination of item–domain associations indicated that participants rated both ease of use items (p1 and p2) positively, suggesting that the smart glasses facilitated observation and provided adequate image clarity. The item reflecting learning and procedural understanding (p3) also received strong agreement, indicating that the device enhanced participants’ comprehension of the catheterization steps. Items related to confidence and efficiency (p4 and p5) showed generally favorable evaluations, with increased confidence reported by participants, although perceptions of efficiency were comparatively lower. Comfort (p6) presented the lowest ratings, pointing to ergonomic limitations during prolonged use. Finally, the global acceptance item (p7) showed that participants were willing to recommend the technology for similar procedures. These relationships are summarized in a conceptual diagram illustrating the organization of domains and items (Fig. 3).

Overall, the use of AR smart glasses was perceived as supportive during the simulated procedure. Participants reported improved procedural understanding, increased confidence, and high visual clarity, which may support more efficient information processing. Engagement and motivation were also reported as favorable, reflecting openness to adopting AR in future training.

In contrast, efficiency and comfort received comparatively lower ratings, suggesting that although AR may support conditions that could facilitate procedural performance, ergonomic adjustments may be necessary for extended or repeated training sessions. These findings are further explored through a conceptual synthesis presented in Table 2.

**Table 2:** Conceptual interpretation of perceived usability findings of augmented-reality smart glasses during the simulated procedure.

Conceptual dimension (domain)	Findings from participants’ responses	Potential implications (hypothesis-generating)
Ease of use (D1) (*p1, p2*)	High agreement that smart glasses facilitated observation (*p1, median 5*) and provided adequate image quality (*p2, median 4.5*)	May support perceptual guidance during early skill acquisition
Learning / procedural understanding (D2) (*p3*)	Participants reported greater perceived understanding of catheterization steps (*median 4.5*)	May support engagement with procedural steps through real-time visualization
Confidence / efficiency (D3) (*p4, p5*)	Increased confidence in performance (*p4, median 4*) and moderate perceptions of procedural efficiency (*p5, median 3.5*)	May be associated with increased self-confidence during task execution and suggest potential applicability in workflow-oriented training contexts
Comfort (D4) (*p6*)	Lower ratings related to comfort (*median 3.*5), indicating ergonomic limitations	Highlights the potential need for ergonomic improvements to support prolonged or repeated use
Global acceptance (*p7*)	Strong intention to recommend smart glasses for similar procedures (*median 4.5*)	Suggests perceived value and openness toward the adoption of AR-supported tools in training contexts

Source: Elaborated by the authors.

The following table presents a conceptual interpretation of the findings, exploring their potential implications for clinical skills training.

## Discussion

This study evaluated the usability and feasibility of AR smart glasses in a simulated, remotely guided clinical procedure. The results indicate that participants perceived the technology as generally usable and supportive, although responses varied across different usability domains. These findings align with previous studies showing that AR can enhance trainees’ experience, provide real-time feedback, and support procedural training in both simulated and clinical contexts^1,5,6^.

Importantly, this study did not assess objective procedural performance or training-related outcomes. Instead, it focused on subjective user’s perceptions, which represent an essential first step in evaluating emerging technologies. These perceptions are consistent with established educational theories and prior literature suggesting potential benefits of such tools. Smart glasses with AR functionality may support procedural training in simulation-based environments. The real-time projection of technical instructions into the learner’s field of view aligns with the principles of experiential learning, in which immediate feedback and hands-on practice are associated with active engagement during skill acquisition. These findings are compatible with theoretical mechanisms described by cognitive load theory^19,20^, suggesting that smart glasses may facilitate interaction with procedural information during the simulation. However, these hypotheses were not directly measured and should be investigated through objective cognitive load assessments in future studies.

The variability in participant experience levels may have influenced the results. More experienced participants may have found the system intuitive, while less experienced individuals may have benefited more from visual guidance. Future studies should include stratified analyses to explore these differences.

The absence of objective performance measures (*e.g.*, procedural time, error rates, and structured assessment) limits the ability to draw conclusions regarding training-related effectiveness. Future research should incorporate these metrics to better evaluate objective training-related outcomes. Other studies have reported potential benefits in simulated thoracostomy training, in which AR-supported telementoring improved communication, confidence, and perceived usefulness among participants^21^. Similarly, AR-based systems have been associated with enhanced procedural guidance and remote supervision, supporting their role in distributed medical education^11,22^.

Nevertheless, the lower scores for comfort and efficiency illustrate the limitations commonly described in AR research, particularly regarding physical ergonomics, weight distribution, heat generation, and visual fatigue^18^. Concerns about usability during prolonged use have also been identified in emergency medicine applications^23^, highlighting the importance of continuous improvements in device construction to enhance comfort and ergonomics. Although audiovisual latency is cited as an acknowledged technical challenge to telementoring^18^, no connectivity issues occurred in this simulation, likely due to the controlled environment and stable network infrastructure.

This study also contributes to the broader discussion regarding remote supervision and equitable access to expert instruction. AR-enabled telementoring may benefit institutions with limited availability of experienced preceptors, supporting more democratized training opportunities. The approach is aligned with global strategies to reduce disparities in healthcare workforce distribution and enhance emergency care capacity^24^.

Regarding the measurement instrument, although it was informed by established frameworks (SUS and TAM), it was not externally validated. High internal consistency alone does not guarantee construct validity, and the lack of validation limits comparability with other studies. This should be considered when interpreting the findings. Additionally, the use of only positively worded items may have introduced acquiescence bias.

This study has several limitations. First, the single-group design without a control condition limits causal inference regarding training-related effects. Second, no objective performance measures, such as procedural accuracy or task completion time, were collected, as the focus was on usability and user’s experience in a simulated setting. Third, the sample was relatively small and homogeneous, which may limit generalizability, although the controlled environment helped ensure consistency across participants. Variability in prior experience may also have influenced perceptions. In addition, the measurement instrument was not externally validated, although it was informed by established frameworks and refined through expert review and preliminary testing. These limitations reinforce the exploratory nature of the findings and highlight the need for controlled comparative studies.

Future research should consider the use of standardized usability tools or hybrid instruments to strengthen validity evidence. The study also presents limitations related to a relatively small and homogeneous sample drawn from a single academic program, which restricts generalizability. Furthermore, the focus of the simulation on a single procedure may not reflect usability across diverse clinical tasks. Longitudinal designs that assess skill retention, transfer of performance to real patients, and comparative effectiveness against traditional simulation-based teaching are needed.

Overall, the findings suggest that AR smart glasses are feasible and acceptable tools for remote-guided simulation, supporting further investigation in controlled comparative studies. With continued advancements in software integration and artificial intelligence, AR may further expand its role in medical education, remote training, and clinical decision support as these technologies continue to evolve.

## Conclusion

This feasibility study suggests that AR smart glasses are acceptable and usable for simulated remote-guided clinical training, with potential applicability in simulation-based procedural training. Further controlled studies incorporating objective learning and performance measures are needed to assess the role of AR technologies in clinical training.

## Data Availability

The datasets used and/or analyzed during the current study are available from the corresponding author upon reasonable request. Shared datasets will be provided in a de-identified format in accordance with institutional ethical requirements.
